# Environment-Friendly and Two-Component Method for Fabrication of Highly Hydrophobic Wood Using Poly(methylhydrogen)siloxane

**DOI:** 10.3390/polym13010124

**Published:** 2020-12-30

**Authors:** Jie Gao, Wensheng Lin, Shumin Lin, Xinxiang Zhang, Wenbin Yang, Ran Li

**Affiliations:** College of Materials Engineering, Fujian Agriculture and Forestry University, Fuzhou 350108, China; gaojiefafu@163.com (J.G.); wensheng0817@163.com (W.L.); lsm176170@163.com (S.L.); xxzhang0106@163.com (X.Z.)

**Keywords:** wood, hydrophobicity, two-component, poly(methylhydrogen)siloxane (PMHS), anti-fouling

## Abstract

Practical application of wood remains a great challenge because of its highly hydrophilic property. In this work, highly hydrophobic wood was produced using an environment-friendly and two-component package method. Poly(methylhydrogen)siloxane (PMHS) and inhibitor played the key role in the hydrophobicity of wood and the assembly process. The two-component package mechanism was discussed in detail. As a result, the water contact angles of the modified wood surface for the radial and cross sections were 139.5° and 152.9°, respectively, which provided the resultant wood high hydrophobicity and dimensional stability. The two-component package method afforded the wood good anti-fouling property and UV-resistance. In addition, the two-component package method could also be applied in functionalization of filter paper for oil/water separation.

## 1. Introduction

The application of wood in outdoor environments is limited due to its highly hydrophilic property, which results in wood deformation, decay, and strength degradation significantly reducing the wood’s service life [1,2,3,4]. Therefore, it is highly desirable to modify the wood. The traditional methods of wood modification include resin and oil treatment [5,6], heat treatment [7,8], surface chemical treatment (including acetylation treatment, grafting of hydrophobic carbon chains, grafting polymers) [9,10,11,12], etc. These traditional treatment methods can slow down the absorption of moisture by wood but cannot fundamentally inhibit the intrusion of moisture into wood. In the recent decade, building a superhydrophobic coating on the surface of a wooden substrate has been considered an effective way to improve the water-repellency of wood [13,14,15,16]. There are many papers reporting the successful preparation of superhydrophobic surfaces on wood. Jia et al. fabricated superhydrophobic wood using nano-SiO_2_ and vinyltriethoxysilane (VTES) by an alkali-driven method. The treated wood has a superhydrophobic property with a water contact angle (WCA) of 156.6° and a sliding angle (SA) of 1.8° [14]. Kong et al. successfully deposited zinc oxide (ZnO) nanorod arrays on the wood surface by a low-temperature hydrothermal method; the WCA of the treated wood can reach 154° [17]. Tu et al. successfully prepared mechanically wear-resistant superhydrophobic wood by using silica, epoxy resin, and FAS as raw materials; the obtained superhydrophobic wood can withstand sandpaper abrasion and knife scraping [18]. In the wood modification methods mentioned above, most of them combine low surface energy materials with rough surface to construct a micro-nano hierarchical structure on the wood surface, which endows the wood with superhydrophobic properties [19,20]. However, the modification process involved in the above methods is too complicated, time-consuming, and expensive. Therefore, it limits their practical application. In fact, for the anti-fouling and water-repellent properties of wood, being highly hydrophobic is sufficient, and it does not need to be superhydrophobic.

Recently, our previous work applied poly(methylhydrogen)siloxane (PMHS) as a hydrophobic modifier to afford wood good hydrophobicity and anti-fouling properties [21]. The modification process of wood was simple, effective, and inexpensive by immerging wood into the modifier solution at room temperature. However, the modifier solution was composed of about 1 wt.% PMHS and 99 wt.% hexane. Hexane is not environment-friendly. The modification of wood by PMHS based on the dehydrogenation between –Si–H of PMHS and –OH groups on wood surface. The –Si–H groups of PMHS possess ultra-high reactivity with –OH and –CH=CH_2_ groups in the presence of Kastredt catalyst. This limited the solvents for modifier solution to be nonpolar, including hexane, tetrahydrofuran, toluene, and so on, which are generally environment-unfriendly.

In this work, an environment-friendly modifier solution was prepared by using ethanol as solvent with a two-component package. The aim of the two-component package is to isolate PMHS from Kastredt catalyst. Component A is composed of PMHS, ethanol, and inhibitor, while component B consisted of ethanol and Kastredt catalyst. The modification of wood can be carried out by mixing two components and then spraying the mixture onto the wood surface. This environmentally friendly and two-component package method afforded wood very high hydrophobicity, UV-resistance, and anti-fouling.

## 2. Materials and Methods

### 2.1. Materials

Ethanol (analytical grade) was purchased from Tianjin Zhiyuan Chemical Reagent Co., Ltd. (Tianjin, China). PMHS with a content of active hydrogen (Si–H) of 1.5% and Kastredt catalyst (platinum-1,3-divinyl-1,1,3,3-tetramethyldisiloxane) with Pt content of 2000 ppm were provided by Chengguang Research Institute of Chemical Industry (Chengdu, China). Inhibitor (1-ethynyl-Cyclohexanol) was obtained from Dongguan Zhongxin Silicone Material Co., Ltd. (Guangzhou, China). The wood samples of Chinese Cunninghamia lanceolata (obtained from Fujian, China) were cut parallel to the grain direction and sawn into blocks of 20 mm × 20 mm, and the thickness of the wood chips was controlled within 12 mm. In addition, all the chemical reagents in this work were used as received without further purification.

### 2.2. Fabrication of Two-Component Modifier Solution

PMHS (0.5 g) was added in ethanol (24.5 g) as the hydrophobic modifier for wood samples, and then inhibitor was added to obtain component A. In addition, Kastredt catalyst was added in ethanol (25 g) to obtain component B. The amount of inhibitor relative to the catalyst was 0%, 14%, 28%, 42%, 56%, and 70%.

### 2.3. Surface Modification of Wood Samples

Prior to the modification treatment, Solution A and B were mixed together in a spray gun to form a spraying solution, which was simply sprayed on wood samples. Finally, the treated samples were dried at room temperature for several minutes and then cured at 80 °C for 1 h.

### 2.4. Characterization

Before characterization, all the samples were washed by ethanol three times and dried at 80 °C for 1 h. The morphologies of the wood samples were characterized using a scanning electron microscope (SEM, ZEISS Z500, Oberkochen, Germany) operating at an acceleration voltage of 20 kV in combination with X-ray spectroscopy (EDS, Genesis, EDAX). Fourier transform infrared spectroscopy (FTIR, Bruker Tensor 27, Billerica, MA, USA) using the KBr pellet method was used to analyze the chemical composition of the as-prepared samples. Changes in the surface chemistry of the wood before and after the PMHS modification by two-component package method was analyzed using X-ray photoelectron spectroscopy (XPS, Thermo Scientific Escalab 250Xi, Waltham, MA, USA). The static WCA and SA of the wood were measured on a commercial contact angle meter (HARKE-SPCA-1, Beijing, China) at room temperature with a distilled water drop volume of 5 μL. In order to characterize the water absorption and dimensional stability of modified wood by two-component package method, the modified wood was tested according to the reported method [10,22].

## 3. Results and Discussion

### 3.1. Hydrophobicity of Wood

**Figure 1:**
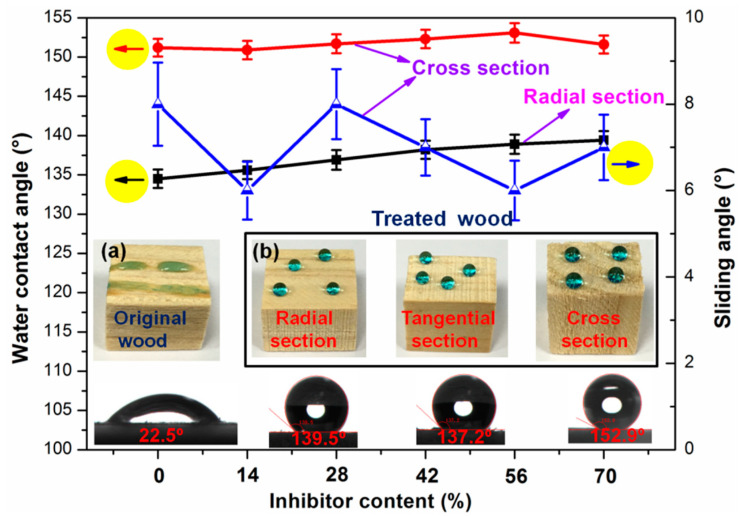
Water contact angle (WCA) and sliding angle (SA) on wood surface at different inhibitor contents. (**a**,**b**) Photographs of water droplets (dyed with methylene blue) on the original and modified wood (radial section, tangential section, and cross section).

PMHS and inhibitor play the key role in adjusting the properties of modifier solution and the modified wood. Therefore, the effect of additional content of PMHS and inhibitor on the hydrophobicity of wood was investigated. In our previous work [21], PMHS content was optimized to be 1.0 wt.% to acquire a desirable hydrophobicity. Figure 1 reveals the effect of inhibitor content on the hydrophobicity of wood at radial- and cross-sections. Without PMHS modification, the water droplet was absorbed totally by wood at cross-section, and the WCA was recorded as 0.0°. As shown in Figure 1a, the water droplet spread well at radial section of unmodified wood, and the WCA was about 22.5°. Figure 1 reveals that PMHS modification significantly improved the hydrophobicity, and the WCA increased slightly with inhibitor content. As shown in Figure 1, without inhibitor, the modification of wood significantly improved the hydrophobicity. However, there are some disadvantages. First, after mixing component A and B, there were many bubbles of hydrogen gas generated within 1 min, and the modifier solution failed quickly. Second, as mixing two components together, –Si–H of PMHS reacted immediately with –OH of ethanol and the reactive –Si–H bonds on PMHS chains were replaced by inert –OCH_2_CH_3_ groups. This is not good for the covalent grafting of PMHS chains onto the wood surface. Inhibitor was very important in prolonging the service life of the modifier solution. The service life of the modifier solution was prolonged to about 3, 5, 7, 10, and 12 min as the inhibitor content (relative to the amount of catalyst) was 14%, 28%, 42%, 56%, and 70%, respectively. According to Figure 1, the wood possessed the best hydrophobicity when the inhibitor content was 56%. The WCA was significantly improved from 22.5° to 139.5° at radial section, while the cross-section was superhydrophobic with the WCA and SA of 152.9° and 6.0°, respectively. In addition, regardless of whether inhibitors are added, the cross-section of the wood was easily superhydrophobic and the sliding angle was less than 10°. This is attributed to the introduction of PMHS chains with very low surface energy onto the wood surface. The better hydrophobicity on cross-section is due to the rougher surface [23,24].

### 3.2. Principle of Two-Component Package

**Figure 2:**
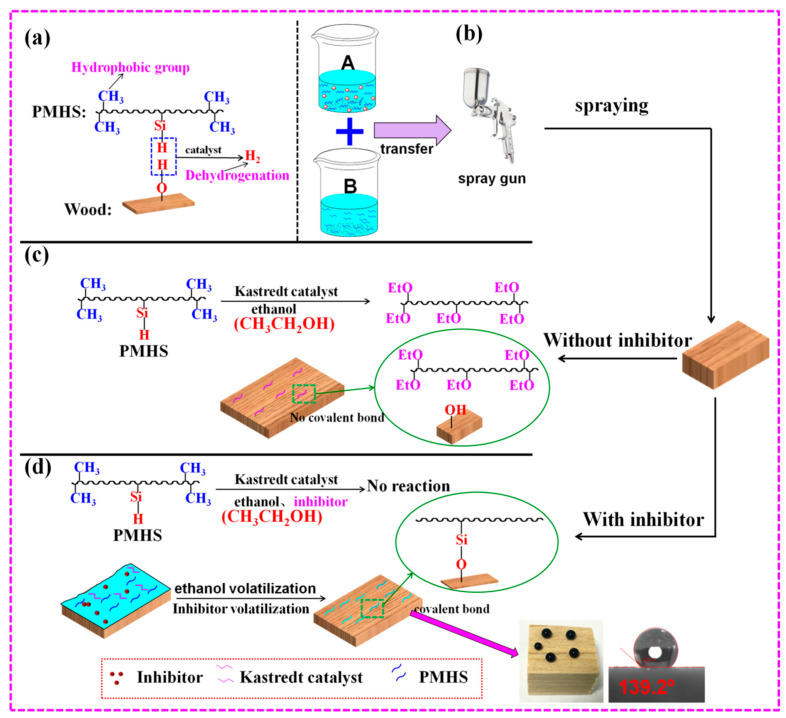
Schematic representation of preparation of hydrophobic wood samples: (**a**) the reaction between PMHS and wood, (**b**) mixing of modified solution, (**c**) without inhibitor, (**d**) with inhibitor.

Figure 1 revealed that PMHS modification of wood by two-component package significantly improves the hydrophobicity of wood, and inhibitor played an important role in control of service life of modifier solution. Schematic representation for preparation of the two-component package modifier solution and its application in hydrophobic modification of wood surface was shown in Figure 2. PMHS is an effective hydrophobic modifier for hydroxyl-containing substrate, such as wood, cotton fabric, and so on. As shown in Figure 2a, PMHS contains lots of hydrophobic methyl groups and reactive –Si–H groups. The –Si–H groups of PMHS possess ultra-high reactivity with hydroxyl groups in the presence of Kastredt catalyst. Before depositing PMHS onto the wood surface, it should be diluted with large amounts of solvent. The solvent should be hydroxyl-free to avoid the reaction between PMHS and solvent. In our previous work, the one-component package was applied to prepare modifier solution by mixing hexane, PMHS, and Kastredt catalyst together. Figure 2b revealed a different two-component package for preparation of modifier solution. Component A was composed of PMHS, ethanol, and inhibitor, while component B consisted of ethanol and Kastredt catalyst. Kastredt catalyst and PMHS were isolated in two different components. As shown in Figure 2b, before wood surface modification, component A and B were mixed together. Then, the mixture was sprayed onto the wood at room temperature. As shown in Figure 2c, without inhibitor, PMHS reacted with ethanol immediately as mixing component A and B together, which resulted in the short service life of modifier solution. Inhibitor can inhibit the activity of Kastredt catalyst, and the inhibition time depends on the addition amount of inhibitor. Therefore, with appropriate amount of inhibitor, no reaction between component A and B happened after mixing them. Furthermore, after spraying the mixture of component A and B onto wood surface, ethanol and inhibitor evaporated from wood surface due to high volatility. After that, the inhibitor lost inhibition effect on the Kastredt catalyst, and PMHS reacted with hydroxyl groups on wood surface by dehydrogenation. Finally, PMHS chains with very low surface energy were grafted onto wood surface, which significantly improved the hydrophobicity of the wood, as shown in Figure 2d.

### 3.3. Change in Chemical Properties of Wood

**Figure 3:**
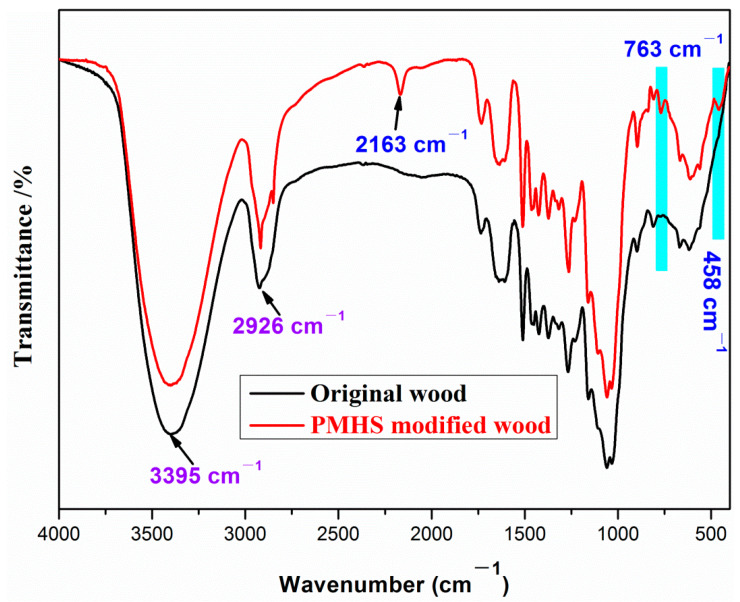
FTIR spectra of the original and poly(methylhydrogen)siloxane (PMHS) modified wood sample.

FTIR characterization was used to observe the changes of surface chemical composition before and after wood modification. FTIR spectra of the original and modified wood samples were recorded and shown in Figure 3. For original wood, there were absorption bands at 3395, 2926, 1740, and 1516 cm^−1^. The absorption bands at 3395 cm^−1^ are attributed to the stretching vibration of the –OH groups [25]. The absorption bands at 2926 cm^−1^ correspond to the stretching vibration of the –CH_3_ groups [26]. In addition, the absorption bands at 1740 cm^−1^ and 1516 cm^−1^ are attributed to the C=O stretching vibration and C=C asymmetric stretching vibration, respectively [21]. For PMHS-modified wood, additional absorption bands appear at 2163, 763, and 458 cm^−1^. The absorption band at 2163 cm^−1^ is attributed to the –Si–H of the PMHS chains [27]. The absorption band at 763 and 458 cm^−1^ is assigned to the Si–O–Si characteristic absorption bands [1,28]. FTIR results indicated that there were PMHS chains on the wood surface.

**Figure 4:**
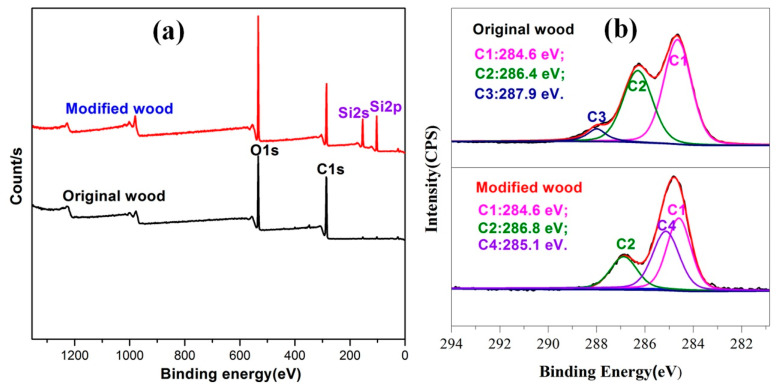
XPS spectra of original and modified wood (**a**) and high-resolution spectra of C1s (**b**).

In addition, through XPS analysis technology, the chemical composition of wood before and after modification and the chemical state of each element were determined. As shown in Figure 4a, there were two characteristic signal peaks in both unmodified and PMHS-modified wood. These two signal peaks were located at 285.08 eV (C1s) and 531.08 eV (O1s) [29]. Moreover, two new signal peaks appeared on PMHS modified wood at positions 103.08 eV (Si2p) and 154.08 eV (Si2s). This indicates that PMHS chains were incorporated on the surface of modified wood. In order to further analyze the chemical composition of the wood surface before and after modification, the XPS peak-differentiation-imitating analysis of C1s was studied. As shown in Figure 4b, there are three functional characteristic groups in original wood (named C1, C2, and C3), which can be attributed to the characteristic signal of cellulose in wood [30]. However, a new peak (C4) at 285.1 eV appeared in the modified wood spectra, and there was no C3 peak. The C4 peak can be attributed to the C–Si (Si–CH_3_) bonds from PMHS chains. In short, XPS results further demonstrate that there were PMHS chains on the wood surface.

### 3.4. Morphological Observation and Elemental Composition Analysis

**Figure 5:**
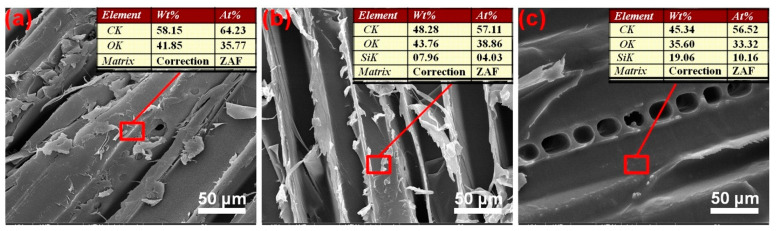
SEM images and EDS analysis of wood treated with different inhibitor contentscontent: original wood (**a**), 42% (**b**), 56% (**c**).

SEM combined with EDS analysis can observe the changes of wood morphology and chemical composition before and after modification. The SEM images of the radial section of the samples are depicted in Figure 5. The original wood surface was smooth and contained a few pipes of tree, as shown in Figure 5a. After surface modification (Figure 5b,c), we could see that the surface topography of wood had no significant changes, which indicated that the two-component package modification method did not influence the microstructure. The surface chemical elemental compositions of the wood before and after surface modification were determined via EDS analysis, and the results are presented as an inset in Figure 5a–c. The EDS analysis of the unmodified wood revealed that it contained C (64.23%) and O (35.77%) atoms, and the Si element was not detected. For the modified wood by two-component package method, the content of Si increased significantly from 4.03 to 10.16% as the inhibitor content increased. This is due to the increase of the inhibitor content, which makes more PMHS chains react to the wood surface, so the silicon content will increase. In short, combining FTIR, XPS, and the SEM-EDS analysis, we could further speculate that the PMHS chains had grafted onto the wood surface. Thus, the wood had good hydrophobicity.

### 3.5. Durability of the Hydrophobic Surfaces

**Figure 6:**
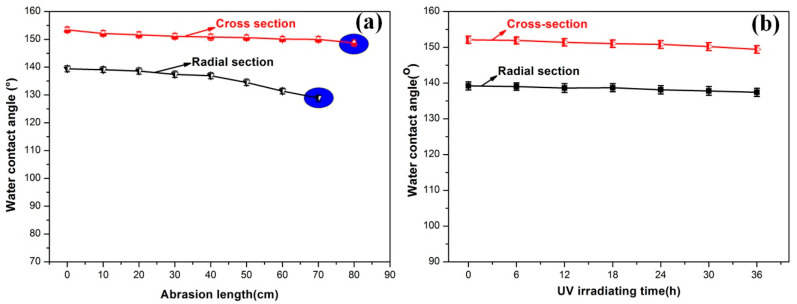
(**a**) WCA variations as a function of the abrasion length; (**b**) the change in WCA of treated wood as a function of UV irradiation time.

To get the application in practice, the hydrophobic surface needs to sustain various types of mechanical damages in real-life conditions. Sandpaper abrasion tests were carried out to evaluate the abrasion durability of the hydrophobic surface of wood. As shown in Figure 6, wood samples (56% inhibitor treated wood) were rubbed with sandpaper (1500 mesh) by loading 20 g weights on the sample. The WCA value of the resultant surfaces was measured at every 20 cm of abrasion length. As can be seen in Figure 6a, when the WCA at radial section and cross section was smaller than 130° and 150°, respectively, the abrasion test was stopped. It was found that the abrasion length of the treated wood at radial and cross section was 60 cm and 70 cm, respectively. This result shows that the modified hydrophobic wood had a certain resistance to mechanical abrasion. This can be attributed to PMHS chains with very low surface energy covalently grafted onto the wood surface.

To further evaluate the durability of the hydrophobic surface, a UV resistance test was performed. As shown in Figure 6b, the prepared wood samples were irradiated under a 48 W UV light with a wavenumber of 356 nm for 0–36 h. It can be clearly seen from the figure that after 36 h UV irradiation, the WCA at cross section and radial section were still larger than 150° and 138°. Moreover, it was observed in the experiment that after UV irradiation, the color change of PMHS-modified wood by two-component package method was significantly decreased compared to the original one. This means that the PMHS modified wood by two-component package method possessed good UV durability.

**Figure 7:**
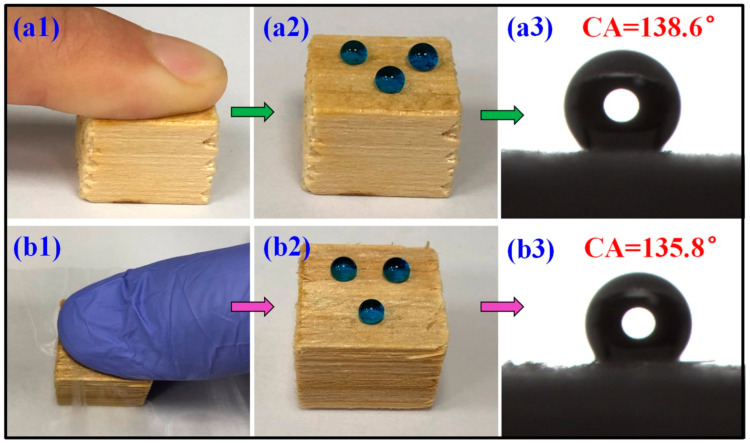
Images of (**a1**–**a3**) finger wiping test and (**b1**–**b3**) tape peeling test.

In addition, in order to qualitatively evaluate the mechanical stability of the hydrophobic film on the modified wood surface, a finger wiping test was carried out, as shown in Figure 7(a1–a3). After finger wiping, the radial surface of the modified wood still had a good hydrophobic effect. The water droplets dyed with methyl blue were spherical on the wiping surface of the modified wood, with a WCA of 138.6°. To further characterize the resistance of the hydrophobic film on the modified wood surface to common damages, a tape peeling test was carried out. As shown in Figure 7(b1–b3, this involved sticking), stick the tape on the surface of the modified wood and pressing it hard several times, then tearing off the tape. The results showed that the surface still had good hydrophobic performance with a WCA of 135.8°.

### 3.6. Water Absorption and ASE Characterization

**Figure 8:**
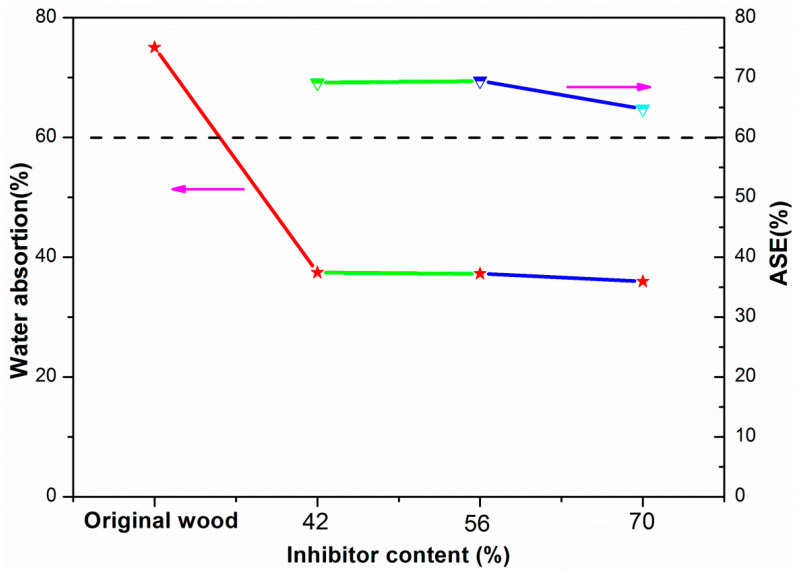
Water absorption rate over 24 h and anti-swelling efficiency (ASE) of original and modified wood at different inhibitor contents.

The water absorption rate of original wood and PMHS-modified wood by two-component package method was tested after being submerged in water for 24 h. As shown in Figure 8, the water absorption rate of original wood was 75.02%, compared with the original wood, the water absorption rate of the modified wood decreased significantly. When the inhibitor content increased to 70%, the treated wood had a very low water absorption value (35.94%).

In order to evaluate the dimensional stability of modified wood, it was characterized by measuring the anti-swelling efficiency (ASE) indices. The higher the value of ASE, the better the dimensional stability [10]. The results are shown in Figure 8. Through calculation, the ASE value of PMHS-modified wood by two-component package method was over 60%. According to Wang’s paper [12], when the ASE value is over 60%, the wood can be considered to have good dimensional stability. The good dimensional stability of modified wood can be attributed to the grafting of the PMHS hydrophobic chain on the surface of the wood in the form of chemical bonds, giving the wood good water-repellency.

### 3.7. Anti-Fouling Property

**Figure 9:**
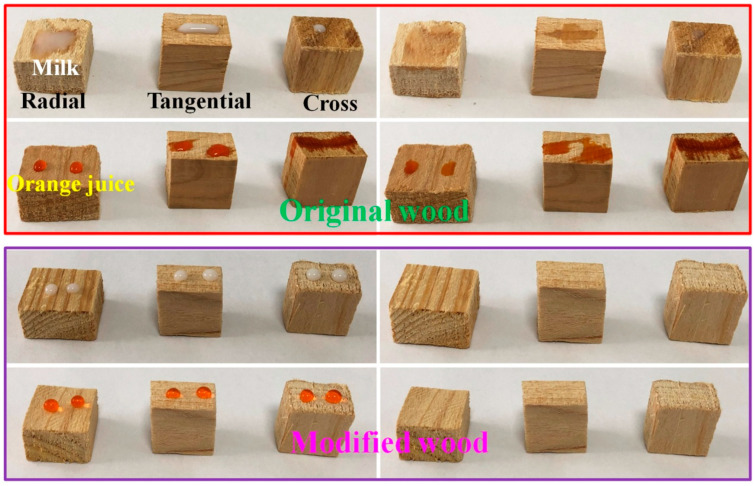
Anti-fouling property of original and modified woods to milk and orange juice.

PMHS modification of wood by the two-component package method significantly improved the hydrophobicity, as shown in Figure 1. However, the radial section of wood did not achieve superhydrophobic effect. This means that PMHS modification of wood by two-component package method had no self-cleaning performance. Nevertheless, for some wood products used indoors, a certain anti-fouling effect is enough. In this study, PMHS is a low surface energy material, which can reduce the surface energy of wood. Consequently, the treated wood had good anti-fouling ability. Figure 9 displayed the images of original and modified woods with milk and orange juice droplets on their surface and the images of them after wiping with paper towel. Obviously, all three sides (radial, tangential, and cross sections) of the original wood were wet by milk and orange juice. This indicates that the original wood was heavily polluted. On the contrary, all three sides of the PMHS-treated wood sample were kept clean and free from pollution. Consequently, it can be inferred that PMHS-treated wood by two-component package method possessed an anti-fouling property towards common liquids.

### 3.8. Potential Application in Oil-Water Separation Area

**Figure 10:**
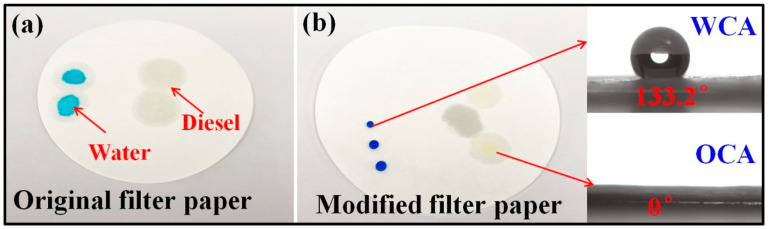
Photographs of (**a**) water droplets with methylene blue as the dye and diesel on filter paper surface before modification; (**b**) water droplets and diesel on filter paper surface after modification.

If a superhydrophobic or hydrophobic membrane layer is constructed on the surface of cotton cloth or filter paper, it can give them good oil/water separation performance [31,32,33]. Taking filter paper as an example, through the two-component package method, a hydrophobic and lipophilic film was prepared on the surface of the filter paper and then the modified filter paper was used as the filter element. As shown in Figure 10a, the original filter paper can absorb water and oil (diesel), the water and diesel drops can completely wet the original filter paper. On the contrary, after PMHS modification by two-component package method, the water droplets were spherical on the surface of the filter paper, and the surface was not wet (the water contact angle increases from 0.0° to 133.2°). Moreover, the drops of diesel completely wet the treated filter paper. This means that the modified filter paper had hydrophobic and lipophilic properties, as shown in Figure 10b. Consequently, the PMHS-modified filter paper can be used in the field of oil-water separation.

**Figure 11:**
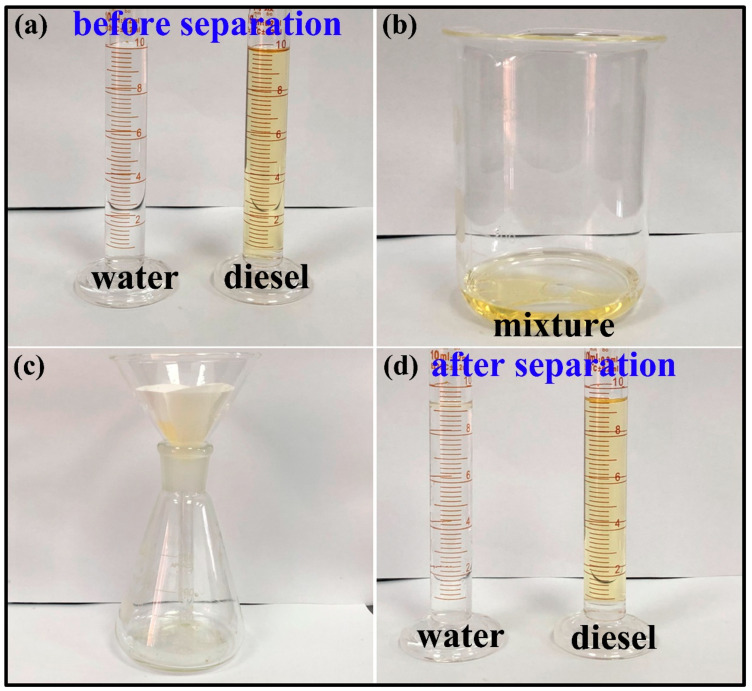
Images of (**a**) volume of water and diesel before separation; (**b**) as-prepared mixtures of 10.0 mL water with 10.0 mL diesel; (**c**) the equipment of separation of oil and water; (**d**) volume of water and diesel after separation.

In order to test the oil-water separation efficiency of the PMHS modified filter paper, a certain amount of water (10 mL) and diesel (10 mL) was mixed and then the mixture was separated by using modified filter paper as shown in Figure 11a,b. As shown in Figure 11c, the oil-water separation device consisted of a conical flask, funnel, and modified filter paper. Then, the oil-water mixture was poured into the oil-water separation device. It can be seen that the diesel wet the hydrophobic filter paper and gradually penetrated through the surface of the hydrophobic filter paper and fell into the conical flask. In contrast, water still remained and collected on the surface of the hydrophobic filter paper. Finally, the water was poured into the measuring cylinder. The results are shown in Figure 11d. The oil-water separation efficiency of the filter material is characterized by calculating the volume ratio of water before and after separation. Through calculation, the separation efficiency of PMHS-modified filter paper can reach up to 94%. In short, PMHS-modified filter paper by two-component method can be applied in oil-water separation area.

## 4. Conclusions

In summary, highly hydrophobic wood with anti-fouling properties was successfully fabricated by a two-component package method. The WCA of the hydrophobic wood could be increased by tuning the amount of inhibitor, reaching a maximum value at 56% inhibitor. The role of the inhibitor is to control the reaction between PMHS, ethanol, and wood, and the PMHS could graft on the wood surface to reduce their surface energy. The two-component package mechanism was proposed to explain the reason for isolated PMHS from the Kastredt catalyst. The two-component package method gave wood with UV-resistance, improved water resistance, and dimensional stability, and gave filter paper good oil/water separation ability.

## Data Availability

Not applicable.

## References

[1] Lu Y., Feng M., Zhan H. (2014). Preparation of sio_2_-wood composites by an ultrasonic-assisted sol–gel technique. Cellulose.

[2] Han X., Yin Y., Zhang Q., Li R., Pu J. (2018). Improved wood properties via two-step grafting with itaconic acid (ia) and nano-sio_2_. Holzforschung.

[3] Niu K., Song K. (2020). Surface coating and interfacial properties of hot-waxed wood using modified polyethylene wax. Prog. Org. Coat..

[4] Huang Y., Feng Q., Ye C., Nair S.S., Yan N. (2020). Incorporation of ligno-cellulose nanofibrils and bark extractives in water-based coatings for improved wood protection. Prog. Org. Coat..

[5] Furuno T., Imamura Y., Kajita H. (2004). The modification of wood by treatment with low molecular weight phenol-formaldehyde resin: A properties enhancement with neutralized phenolic-resin and resin penetration into wood cell walls. Wood Sci. Technol..

[6] Mourant D., Yang D.Q., Riedl B., Roy C. (2008). Mechanical properties of wood treated with pf-pyrolytic oil resins. Holz Roh Werkst.

[7] Hakkou M., Petrissans M., Zoulalian A., Gerardin P. (2005). Investigation of wood wettability changes during heat treatment on the basis of chemical analysis. Polym. Degrad. Stabil..

[8] Okon K.E., Lin F., Lin X., Chen C., Chen Y., Huang B. (2018). Modification of Chinese fir (*Cunninghamia lanceolata* L.) wood by silicone oil heat treatment with micro-wave pretreatment. Eur. J. Wood Wood Prod..

[9] Matsunaga M., Hewage D.C., Kataoka Y., Ishikawa A., Kobayashi M., Kiguchi M. (2016). Acetylation of wood using supercritical carbon dioxide. J. Trop. For. Sci..

[10] Wang K., Dong Y., Yan Y., Zhang W., Qi C., Han C., Li J., Zhang S. (2017). Highly hydrophobic and self-cleaning bulk wood prepared by grafting long-chain alkyl onto wood cell walls. Wood Sci. Technol..

[11] Huang X., Kocaefe D., Kocaefe Y.S., Pichette A. (2018). Combined effect of acetylation and heat treatment on the physical, mechanical and biological behavior of jack pine (*Pinus banksiana*) wood. Eur. J. Wood Wood Prod..

[12] Wang K., Dong Y., Yan Y., Zhang S., Li J. (2018). Improving dimensional stability and durability of wood polymer composites by grafting polystyrene onto wood cell walls. Polym. Compos..

[13] Chen Y., Wang H., Yao Q., Fan B., Wang C., Xiong Y., Jin C., Sun Q. (2017). Biomimetic taro leaf-like films decorated on wood surfaces using soft lithography for superparamagnetic and superhydrophobic performance. J. Mater. Sci..

[14] Jia S., Chen H., Luo S., Qing Y., Deng S., Yan N., Wu Y. (2018). One-step approach to prepare superhydrophobic wood with enhanced mechanical and chemical durability: Driving of alkali. Appl. Surf. Sci..

[15] Tu K., Wang X., Kong L., Guan H. (2018). Facile preparation of mechanically durable, self-healing and multifunctional superhydrophobic surfaces on solid wood. Mater. Des..

[16] Jia S., Lu X., Luo S., Qing Y., Yan N., Wu Y. (2018). Efficiently texturing hierarchical epoxy layer for smart superhydrophobic surfaces with excellent durability and exceptional stability exposed to fire. Chem. Eng. J..

[17] Kong L., Tu K., Guan H., Wang X. (2017). Growth of high-density zno nanorods on wood with enhanced photostability, flame retardancy and water repellency. Appl. Surf. Sci..

[18] Tu K., Wang X., Kong L., Chang H., Liu J. (2016). Fabrication of robust, damage-tolerant superhydrophobic coatings on naturally micro-grooved wood surfaces. RSC Adv..

[19] Feng L., Li S., Li Y., Li H., Zhang L., Zhai J., Song Y., Liu B., Jiang L., Zhu D. (2002). Super-hydrophobic surfaces: From natural to artificial. Adv. Mater..

[20] Feng L., Li S., Li H., Zhai J., Song Y., Jiang L., Zhu D. (2002). Super-hydrophobic surface of aligned polyacrylonitrile nanofibers. Angew. Chem..

[21] Lin W., Huang Y., Li J., Liu Z., Yang W., Li R., Chen H., Zhang X. (2018). Preparation of highly hydrophobic and anti-fouling wood using poly(methylhydrogen)siloxane. Cellulose.

[22] Jiang J., Cao J., Wang W. (2018). Characteristics of wood-silica composites influenced by the ph value of silica sols. Holzforschung.

[23] Liu M., Qing Y., Wu Y., Liang J., Luo S. (2015). Facile fabrication of superhydrophobic surfaces on wood substrates via a one-step hydrothermal process. Appl. Surf. Sci..

[24] Wu Y., Jia S., Wang S., Qing Y., Yan N., Wang Q., Meng T. (2017). A facile and novel emulsion for efficient and convenient fabrication of durable superhydrophobic materials. Chem. Eng. J..

[25] Wang S., Liu C., Liu G., Zhang M., Li J., Wang C. (2011). Fabrication of superhydrophobic wood surface by a sol–gel process. Appl. Surf. Sci..

[26] Lin W., Zhang X., Cai Q., Yang W., Chen H. (2020). Dehydrogenation-driven assembly of transparent and durable superhydrophobic ormosil coatings on cellulose-based substrates. Cellulose.

[27] Brassard J., Sarkar D.K., Perron J. (2011). Synthesis of monodisperse fluorinated silica nanoparticles and their superhydrophobic thin films. ACS Appl. Mater. Interfaces.

[28] Latthe S.S., Liu S., Terashima C., Nakata K., Fujishima A. (2014). Transparent, adherent, and photocatalytic sio_2_-tio_2_ coatings on polycarbonate for self-cleaning applications. Coatings.

[29] Poaty B., Riedl B., Blanchet P., Blanchard V., Stafford L. (2013). Improved water repellency of black spruce wood surfaces after treatment in carbon tetrafluoride plasmas. Wood Sci. Technol..

[30] Kumar A., Richter J., Tywoniak J., Hajek P., Adamopoulos S., Segedin U., Petric M. (2017). Surface modification of norway spruce wood by octadecyltrichlorosilane (ots) nanosol by dipping and water vapour diffusion properties of the ots-modified wood. Holzforschung.

[31] Zhou C., Chen Z., Yang H., Hou K., Zeng X., Zheng Y., Cheng J. (2017). Nature-inspired strategy toward superhydrophobic fabrics for versatile oil/water separation. ACS Appl. Mater. Interfaces.

[32] Mai Z., Shu X., Li G., Chen D., Zhang H. (2019). One-step fabrication of flexible, durable and fluorine-free superhydrophobic cotton fabrics for efficient oil/water separation. Cellulose.

[33] Li K., Chen W., Wu W., Pan Z., Liang Z., Gan J. (2020). Facile fabrication of superhydrophilic/underwater superoleophobic polyvinyl acetate/sodium silicate composite coating for the effective water/oil separation and the study on the anti-fouling property, durability and separation mechanism. Prog. Org. Coat.

